# Morphology of the immature stages of *Dasyhelea silvatica* Wang, Zhang & Yu with redescriptions of adults (Diptera, Ceratopogonidae)

**DOI:** 10.3897/zookeys.961.53882

**Published:** 2020-08-19

**Authors:** Xue Lu, Chen Duan, Yuan Ning, Xiao Hong Jiang, Xiao Hui Hou

**Affiliations:** 1 Zunyi Medical University, Zunyi 563000, Guizhou Province, China Zunyi Medical University Zunyi China

**Keywords:** Adult, aquatic, biting midge, fourth instar larva, pupa

## Abstract

The immatures of the biting midge *Dasyhelea silvatica* are described and illustrated for the first time and a complete description of the adult male and female are provided using scanning electron and compound microscopes. The specimens were collected from flooded soil near a pond in Guizhou Province, China, and reared in the laboratory.

## Introduction

Biting midges of the genus *Dasyhelea* Kieffer, 1911 (Diptera, Ceratopogonidae) are a large and complex group of Ceratopogonidae with diverse morphology and biology, and are cosmopolitan in distribution except in Antarctica (Grogan and Wieners 2006). At present there are 192 extant species of *Dasyhelea* in China (Duan et al. 2019; Nie et al. 2019), but only eight of these species are known by their immature stages (Yu et al. 2005, 2013; Duan et al. 2019). This may be an indication that China has been under-sampled historically compared to other countries. Therefore, efforts were made in order to study the immature stages of biting midges in China. During a recent entomological survey carried out in the vicinity of Xiaojiawan, a village in the Guizhou Province, immature specimens of *Dasyhelea silvatica* Wang, Zhang & Yu, 2014 were collected. The purpose of this paper is to provide a complete description, with illustrations, of the fourth instar larva and pupa of *D. silvatica* and a redescription of the adult male and female using a compound and a scanning electron microscope (LM and SEM).

## Materials and methods

Larvae and pupae of *D. silvatica* were collected from flooded soil in Xiaojiawan, Guizhou Province in 2018, using a small shovel, and transferred to the laboratory. The larvae were individually placed in 24-well plates and fed with a sterile nutrient solution of *Chlorella*. Once they pupated, they were isolated in ampoule bottles on filter paper with sugar water. They were reared in an environmental chamber maintained at a temperature of 28 ± 2 °C, a relative humidity of 75 ± 2%, and a photoperiod of 12 h light and 12 h dark, and observed daily until adult emergence. The emergent adults, whole larvae, and pupae were preserved in ethanol at each stage. The specimens were mounted in Canada balsam following the technique described by Yu et al. (2005).

For the SEM study, one larva of *D. silvatica* was prepared following the technique of Ronderos et al. (2000, 2008). Ink illustrations were made using an attached camera lucida. Photographs were taken with a digital system adapted to an Olympus BX43 with a digital camera DP26. The studied material was deposited in the Insect Collection, Zunyi Medical University, Guizhou Province, China (**ICZU**). The morphological terms and identification methods of larvae, pupae and adults used follow Díaz et al. (2013), Borkent (2014) and Yu et al. (2005). The abbreviations used in this paper follow Duan et al. (2019).

## Results

### Dasyhelea silvatica

Taxon classificationAnimaliaDipteraCeratopogonidae

Wang, Zhang & Yu, 2014

260366E4-6EBF-5F72-B1CD-20E153EDD77E

Figures 1 , 2 , 3

Dasyhelea (Dasyhelea) silvatica Wang, Zhang & Yu, 2014: 312 (male and female, China).

#### Material examined.

3 males with pupal exuviae, 4 females with pupal exuviae, 3 fourth instar larvae, 2 larval exuviae. Xiaojiawan Village, Xinpu New District, Zunyi City, Guizhou Province, China, 27°43'22.83"N, 107°04'27.62"E, 7.VII.2018, alt. 866 m, Chen Duan leg. 2 larvae examined by SEM. Same data as above.

#### Descriptions.

**Figure 1.**
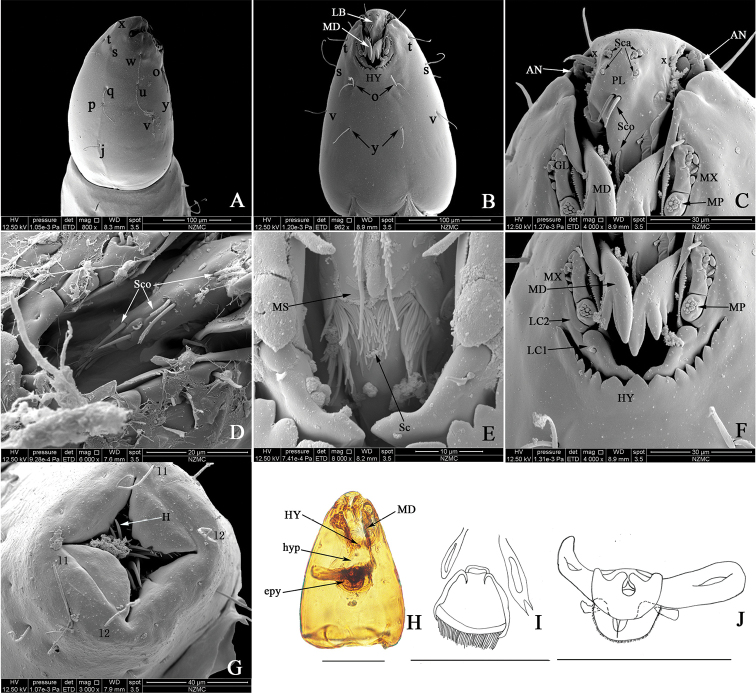
*Dasyhela silvatica* Wang, Zhang & Yu, fourth instar larva **A** chaetotaxy on the head capsule, lateral view (SEM) **B** chaetotaxy on the head capsule, ventral view **C** detail of labrum **D** sensilla coeloconica **E** detail of scopae **F** detail of mouthparts **G** caudal segment **H** head, ventral view **I** hypopharynx **J** epipharynx. Abbreviations: antenna (**AN**); epipharynx (**epy**); galeolacinia (**GL**); hypostoma (**HY**); hooks (**H**); hypopharynx (**hyp**); labrum (**LB**); lacinial sclerite 1 (**LC1**); lacinial sclerite 2 (**LC2**); mandible (**MD**); maxilla (**MX**); maxillary palpus (**MP**); messors (**MS**);palatum (**PL**); sensilla coeloconica (**Sco**); sensilla campaniformia (**Sca**); scopae (**Sc**); first lateral setae (**l1**); second lateral setae (**l2**). Scale bars: 0.1 mm (**G–I**).

**Fourth instar larva** (Fig. 1A–J). Head capsule light brown, long, thin (Fig. 1A, B); chaetotaxy as in Fig. 1A. HL 0.25–0.27 (0.26, *N* = 2) mm; HW 0.16–0.17 (0.16, *N* = 2) mm; HR 1.56–1.58 (1.57, *N* = 2); SGW 0.07–0.08 (0.08, *N* = 2) mm; SGR 0.50–0.52 (0.49, *N* = 2). Antenna (Fig. 1C) short, cylindrical. Anterior portion of palatum (Fig. 1C) with four pairs of campaniformia sensilla; posterior portion with three pairs of coeloconica sensilla, two simple, one serrate (Fig. 1C, D); messors (Fig. 1E) well developed, stout, bisegment; scopae (Fig. 1E) well developed with elongate, strong pointed teeth. Mandible (Fig. 1B, C, F) stout, with four teeth, apical tooth more elongated, proximal tooth minute; MDL 0.07 (*N* = 2) mm, MDW 0.01 (*N* = 2) mm. Maxilla (Fig. 1C, F) well sclerotized; galeolacinia (Fig. 1C) with concentrated flap-like papillae, short seta; maxillary palpus (Fig. 1C, F) cylindrical, with seven or eight apical papillae. Hypostoma (Fig. 1F) with three large mesal teeth, flanked with four strong, lanceolate lateral teeth each side. Lacinial sclerite 1 with one seta; lacinial sclerite 2 without seta (Fig. 1F). Epipharynx (Fig. 1H–J) strongly sclerotized, dorsal comb moderately wide, round, subequally elongate, the middle of trailing edge with a large number of spines on the semicircular transparent membranous structure; lateral arms stout, elongate, with two auxiliary sclerites; LAW 0.10–0.13 (0.12, *N* = 2) mm, DCW 0.03–0.04 (0.04, *N* = 3). Hypopharynx (Fig. 1H, I) stout, heavily sclerotized, posterior comb straight with fringe, labium sclerotized. Thoracic pigmentation diffused, pale brown. Abdominal segments whitish, with diffused pale brown pigmentation. Caudal segment (Fig. 1G) with long stout hooks with pointed tips.

**Figure 2.**
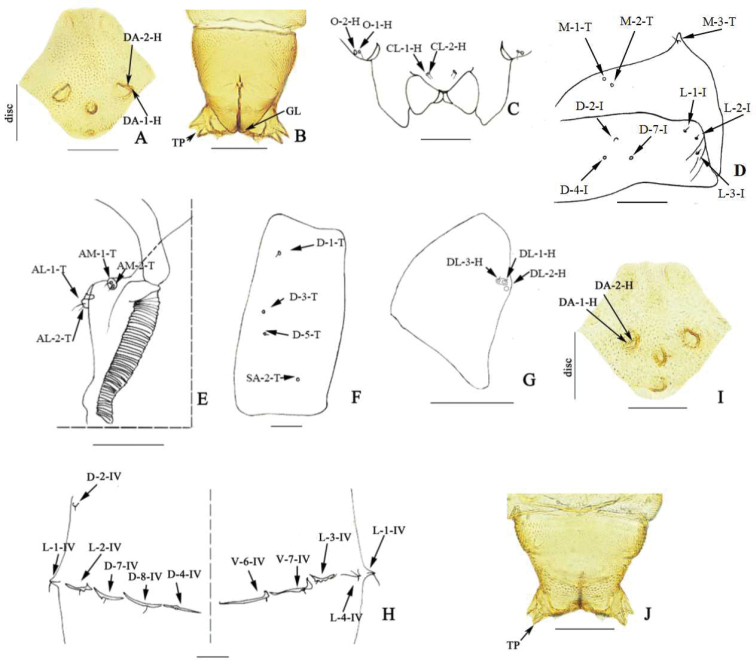
*Dasyhelea silvatica* Wang, Zhang & Yu. Male pupa (**A–H**), female pupa (**I, J**) **A, I** dorsal apotome **B** segment 9 **C** clypeal/labral sensilla and ocular sensilla **D** metathoracic sensilla, and first abdominal segment **E** anterolateral and anteromedial sensilla **F** dorsal and supra-alar sensilla **G** dorsolateral cephalic sclerite sensilla **H** segment 4 **J** segment 9. Abbreviations: Anterolateral sensilla (AL-1-T, AL-2-T, AL-3-T); clypeal/labral sensilla (CL-1-H, CL-2-H); dorsal apotome sensilla (DA-1-H, DA-2-H); dorsolateral cephalic sclerite sensilla (DL-1-H, DL-2-H, DL-3-H); dorsal setae (D-1-T, D-2-T, D-3-T); dorsal sensilla of segment 9 (D-5-IX); methatoracic sensilla (M-2-T, M-3-T); ocular sensilla (O-1-H, O-2-H) respiratory organ (RO); tergite 1 sensilla (D-2-I, D-4-I, D-7-I, L-1-I, L-2-I, L-3-I); genital lobe (**GL**); terminal process (**TP**). Scale bars: 0.1 mm.

**Pupa** (Fig. 2A–J). ***Male*.** Total length 2.51–2.71 (2.63, *N* = 3) mm. General coloration of exuviae pale brown. Head: dorsal apotome (Fig. 2A) 2.15 × broader than long, apex rounded, surface covered with brown rounded tubercles, anterior margin straight, lateral margin smooth, with three anterior wrinkles; apotome sensilla (Fig. 2A): DA-1-H elongate, thin seta, insert on well-developed tubercle, DA-2-H sensillum campaniform at base; disc surface covered by stout, rounded spinules; DAL 0.12–0.13 (0.13, *N* = 2) mm, DAW 0.26–0.29 (0.28, *N* = 2) mm, DAW/DAL 2.15–2.25. Respiratory organ apex dark brown, 7.6 × longer than broad, with circular fold, 12 apical, five lateral pores; ROL 0.22–0.25 (0.23, *N* = 3) mm, ROW 0.03 (0.03, *N* = 3) mm; pedicel pale brown, short, length 0.01 mm, ROP/ROL 0.05–0.07 (0.05, *N* = 3). Mouthparts with mandible, lacinia absent; two clypeal/labrals (Fig. 2C), CL-1-H and CL-2-H medium-sized, thin setae; two ocular sensilla, O-1-H long, thin seta, O-2-H campaniform sensillum (Fig. 2C); metathoracic sensilla (Fig. 2D): M-1-T and M-2-T campaniform sensilla; M-3-T long, thin seta. Tergite 1 (Fig. 2D) setae as follows: D-2-I papilla; D-4-I, D-7-I campaniform sensilla; L-1-I, L-2-I, L-3-I medium-sized, thin setae; two anterolateral sensilla (Fig. 2E): AL-1-T long, thin seta, AL-2-T medium-sized, thin seta; two anteromedial sensilla (Fig. 2E): AM-1-T, AM-2-T medium-sized, thin setae. Cephalothorax surface with small rounded tubercles, cephalothorax length 0.78–0.83 (0.80, *N* = 3) mm, width 0.59–0.64 (0.62, *N* = 3) mm. Cephalothoracic sensilla as follows (Fig. 2F): three dorsal setae, D-1-T and D-5-T short, thin setae, D-3-T campaniform sensillum, SA-2-T supra-alar campaniform sensillum. Cephalothoracic sensilla as follows: three dorsolateral cephalic sclerite sensilla (Fig. 2G), DL-1-H and DL-3-H medium-sized, thin setae, DL-2-H campaniform sensillum. Abdomen covered with short, stout spinules on anterior, posterior margin. Segment 4 (Fig. 2H) with sensillar pattern as follows: D-2-IV short, stout seta; D-4-IV and D-7-IV campaniform sensilla, D-8-IV long, thin setae, all located on flattened tubercles; L-1-IV long, stout seta, L-2-IV short, thin seta, L-3-IV short, stout seta, L-4-IV short, thin seta, all located on triangular tubercles; V-6-IV and V-7-IV short, stout setae, also located on flattened tubercles. Segment 9 (Fig. 2B) 1.01 × longer than wide, length 0.20–0.22 (0.21, *N* = 3) mm, width 0.21–0.22 (0.22, *N* = 3) mm; ventral and dorsal surfaces with many spinules; TP triangular, elongated, acute, length 0.03 (0.03, *N* = 3) mm.

***Female*.** Similar to male with usual sexual differences. General coloration of exuviae pale brown, except dorsolateral cephalic sclerite brown. Dorsal apotome (Fig. 2I), DAL 0.12–0.13 (0.13, *N* = 2) mm, DAW 0.26–0.29 (0.27, *N* = 2) mm, DAW/DAL 2.14–2.26 (2.20, *N* = 2). Cephalothorax length 0.83–1.07 (0.95, *N* = 2) mm, width 0.66–0.80 (0.72, *N* = 2) mm. ROL 0.23–0.27 (0.25, *N* = 2) mm, ROW 0.03 (0.03, *N* = 2) mm; pedicel length 0.01 (*N* = 2) mm, ROP/ROL 0.04 (0.04, *N* = 2). Segment 9 (Fig. 2J) length 0.18–0.23 (0.21, *N* = 2) mm, width 0.22–0.23 (0.23, *N* = 2) mm; ventral surface with many spicules, single funnel-like structure medially. TP triangular, elongated, pointed (Fig. 2J).

#### Redescription of adults

**Figure 3.**
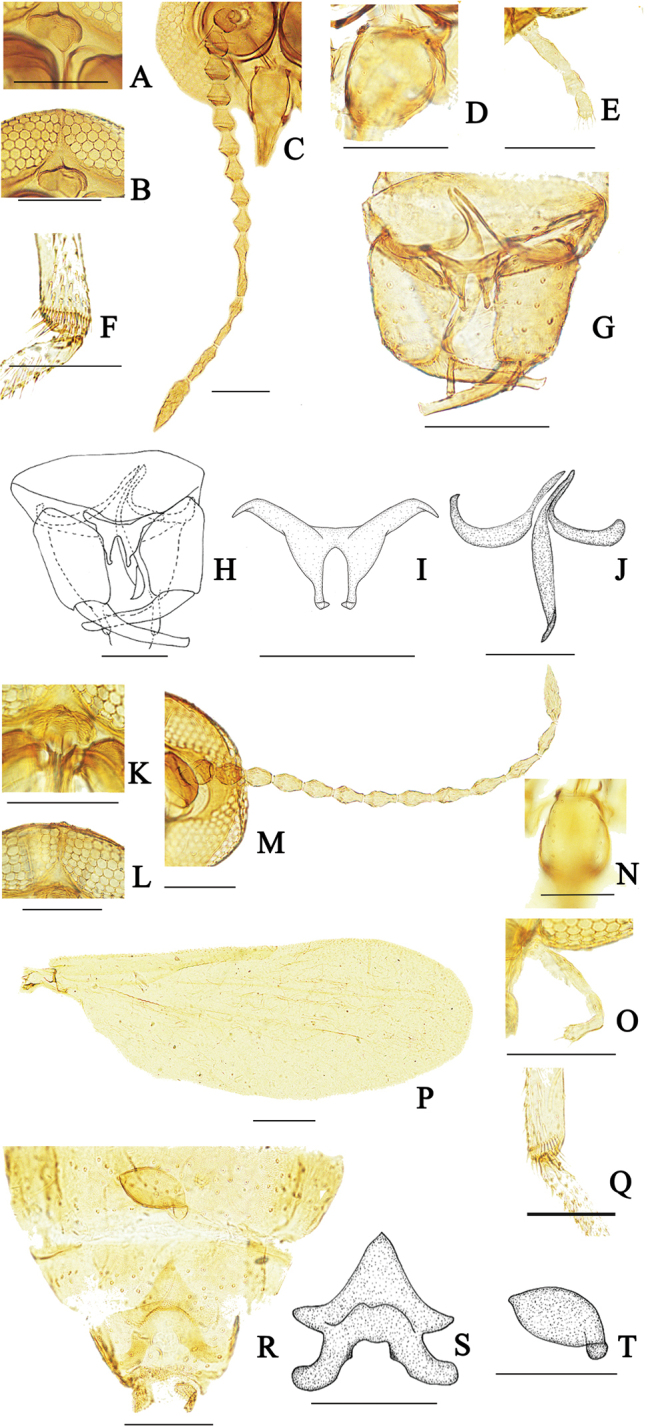
*Dasyhelea silvatica* Wang, Zhang & Yu. Male adult (**A–J**), female adult (**K–T**) **A, K** frontal sclerite, anterior view **B, L** eyes contiguous, anterior view **C, M** flagellomeres, anterior view **D, N** clypeus, anterior view **E, O** palpus, anterior view **F** metatibial distae comb **G** genitalia, ventral view **H** genitalia, ventral view **I** aedeagus **J** paramere **P** wing **Q** hind tibial comb **R** subgenital plate and spermatheca, ventral view **S** subgenital plate, ventral view **T** spermatheca, ventral view. Scale bars: 0.1 mm.

(Fig. 3A–T). ***Male*** (Fig. 3A–J). ***Head*.** Brown. Frontal sclerite nearly round, with long, slender ventral projection (Fig. 3A). Eyes (Fig. 3B) contiguous, abutting medially for length of 1.0 ommatidia, with interfacetal hairs. Antennal flagellum (Fig. 3C) brown, flagellomere 13 with apical projection; AR 1.23. Clypeus (Fig. 3D) with five pairs of setae. Palpus (Fig. 3E) brown; third segment slender, PR 2.67, the length almost the sum of the fourth and fifth segments. Lengths of palpus segments in ratio of 10: 15: 30: 18: 17.

***Thorax*.** Scutum shallow, scutellum yellow, with ten stout setae. Legs light brown, hind tibial comb with seven spines (Fig. 3F); foreleg TR 2.30, midleg TR 2.40, hind leg TR 2.46. Wing length 1.23 mm, width 0.40 mm, CR 0.50; wing membrane hyaline, densely covered with microtrichia, cubital fork at same level of distal portion of second radial cell. Halter light brown.

***Abdomen*.** Brown. Genitalia: tergite 9 nearly trapezoidal with prominent, long with apical stout seta, apico-lateral processes. Posteromedial margin of sternite 9 with inconspicuous projection, Gonocoxite stout, 1.73 X longer than greatest width, gonostylus slender (Fig. 3G, H). Aedeagus (Fig. 3G, I) without median process, posterolateral arm symmetry, each arm tapers from the proximal portion to the distal portion, apex of arm curving inwards, arch high. Parameres (Fig. 3G, J) separate, with median lobe short, thin, lateral lobe curve, stout gradually.

***Female*** (Fig. 3K–T). ***Head*.** Frontal sclerite oval, with long, slender ventral projection (Fig. 3K). Eyes (Fig. 3I) contiguous. Antennal flagellum (Fig. 3M) brown, with sculpture, flagellomere 13 with apical projection; AR 1.01. Clypeus (Fig. 3N) with five pairs of setae. Palpus (Fig. 3O) brown; third segment slender, PR 2.70, lengths of palpus segments in ratio of 3: 6: 14: 6: 8.

***Thorax*.** Wing length 1.29–1.34 (1.22, *N* = 2) mm, width 0.51–0.54 (0.53, *N* = 2) mm, CR 0.48 (Fig. 3P). Hind tibial comb with ten spines (Fig. 3Q); foreleg TR 2.02, midleg TR 2.30, hind leg TR 2.38.

***Abdomen*.** Similar to male. Subgenital plate (Fig. 3R, S) star-shaped, arch high, inner arch with a pair of processes. Spermatheca long ovoid (Fig. 3R, T), diameter 82 μm, neck long, curved, length 14.0 μm.

#### Distribution.

China (Fujian Province, Guizhou Province).

## Discussion

*Dasyhelea silvatica* belongs to the subgenus D. (Dasyhelea) (Wang et al. 2014). The fourth instar larva of *D. silvatica* is very similar to its congeners *D. azteca* Huerta & Grogan by virtue of the antenna being short, the lateral arms of the epipharynx stout and the anterior portion of palatum with four pairs of campaniformia sensilla, but it can be distinguished from the former by the posterior portion of palatum bearing two pairs of coeloconica sensilla, the MD with three teeth, and the MP with only three small papillae. In addition, the fourth instar larva of *D. silvatica* is also similar to that of *D. flavifrons* (Guérin-Méneville), recently described by Díaz et al. (2019) with the brown head capsule, the posterior portion of palatum with four pairs of campaniformia sensilla and three pairs of coeloconica sensilla, the posterior comb of the hypopharynx has a fringe; however, *D. flavifrons* differs by the galeolacinia with 5–6 papillae and the MP with only three or four papillae.

The common characteristics of the pupae of *D. silvatica* and *D. azteca* are as follows: the small rounded tubercles on cephalothorax; the surface of dorsal apotome also has rounded tubercles; the abdominal segments covered with spinules, and the sensilla of the fourth abdominal segment are all located on flattened tubercles. But the pupa of *D. azteca* differs from *D. silvatica* by having a single ocular sensillum, the exuviae is brown in general coloration, and the RO has 22–24 apical and three or four lateral pores, and without a pedicel. The pupa of *D. silvatica* is similar to *D. flavifrons* by virtue of the three dorsolateral cephalic sclerite sensilla and the tergite of the first abdominal segment with the L-1-IV represented by a long and stout seta, but the one ocular sensilla, the RO bearing 14–16 apical and four or five lateral pores, anterolateral sensilla with AL-1-T, AL-2-T long, thin seta and AL-3-T short, stout seta distinguish from *D. silvatica*. Furthermore, the pupa of *D. silvatica* is similar to that of *Dasyhelea eloyi* Díaz & Ronderos, 2013 with small rounded tubercles on cephalothorax surface and the RO with scale-like spines, but the latter has 6–8 lateral pores compared to *D. silvatica* with 5 lateral pores.

Duan et al. (2019) described the larvae and pupae of *D. alula* collected in the same small wetland as *D. silvatica*. The fourth instar larva of *D. silvatica* shows similarities with that of *D. alula* in the rear comb of the hypopharynx with a fringe and two auxiliary sclerites on the lateral arms of the epipharynx. However, the fourth instar larva of *D. alula* is distinctly distinguished by its head capsule being yellowish, short, and wide; three teeth of the mandible are the same size; and hypostoma has the mesal portion smooth. The pupa of *D. alula* differs in the smaller total length (1.97 mm); RO having 7–8 apical and three lateral pores; anterolateral sensilla with AL-1-T medium-sized, thin seta and AL-3-T short, stout seta; the tergite I without L-2-I and L-3-I setae.

Finally, we found a semicircular, transparent membrane, strongly varying in shape, present at the trailing edge of the epipharynx of *D. silvatica*.

## Supplementary Material

XML Treatment for Dasyhelea silvatica
